# Supplementary material for: Implementing a new clinical pathway in a non-receptive context: Mixed methods evaluation of a new fracture pathway for older people in a hospital Trust in the West Midlands, UK
Source: PLoS One. 2021 Feb 22;16(2):e0247455. doi: 10.1371/journal.pone.0247455 (PMC7899317; doi:10.1371/journal.pone.0247455)
Supplement: S1 File — (PDF) [file pone.0247455.s001.pdf]

HECTOR study

Interview topic guide

Introduction

- What's your role in relation to HECTOR?
- What was your understanding of HECTOR when you joined the team? (prompt – booklet, training, database (TARN) etc.)
- Has this changed over time?

Overview

- What is your overall sense of the value of HECTOR?

Development

- Why was HECTOR needed?
- Do you think HECTOR is still needed?
- Has the need for HECTOR changed?

Familiarisation

- What training have you (those who use it) received?
- How effective do you think the training has been?
- What should be considered going forward with regards to training?

Implementation

- Do you think HECTOR needs to be/is being adapted in practice?
    - Do you use HECTOR in ways not originally intended?
    - Are you able to give feedback on how it works in practice?
    - Do you think the feedback would make a difference?

Impact on your work

- How has HECTOR altered the way you (other who use it) work?
    - Has HECTOR changed your working relationships between individuals?

Impact on Patients/Staff

- What do you see as the impacts of HECTOR upon patients?
    - Are there positive impacts? Do you use HECTOR in ways not originally intended?
    - Do you feel there are any negative impacts
    -
- What do you see as the impacts of HECTOR on staff?
    - Are there positive impacts? Do you use HECTOR in ways not originally intended?
    - Do you feel there are any negative impacts
    - Has HECTOR lead to changes in practice that were not expected – positive/negative

Leadership

- What do you feel has been the impact of the leadership of HECTOR upon its development and implementation.

Is there anything that you want to raise, or think that might help my research which I have not yet covered.
